# A Model of DENV-3 Infection That Recapitulates Severe Disease and Highlights the Importance of IFN-γ in Host Resistance to Infection

**DOI:** 10.1371/journal.pntd.0001663

**Published:** 2012-05-29

**Authors:** Vivian V. Costa, Caio T. Fagundes, Deborah F. Valadão, Daniel Cisalpino, Ana Carolina F. Dias, Kátia D. Silveira, Lucas M. Kangussu, Thiago V. Ávila, Maria Rosa Q. Bonfim, Daniela Bonaventura, Tarcília A. Silva, Lirlândia P. Sousa, Milene A. Rachid, Leda Q. Vieira, Gustavo B. Menezes, Ana Maria de Paula, Alena Atrasheuskaya, George Ignatyev, Mauro M. Teixeira, Danielle G. Souza

**Affiliations:** 1 Laboratório de Interação Microrganismo-Hospedeiro, Departamento de Microbiologia, Instituto de Ciências Biológicas, Universidade Federal de Minas Gerais, Belo Horizonte, Brazil; 2 Imunofarmacologia, Departamento de Bioquímica e Imunologia, Instituto de Ciências Biológicas, Universidade Federal de Minas Gerais, Belo Horizonte, Brazil; 3 Laboratório de Farmacologia Cardiovascular, Departamento de Fisiologia e Farmacologia, Instituto de Ciências Biológicas, Universidade Federal de Minas Gerais, Belo Horizonte, Brazil; 4 Departamento de Patologia Oral, Faculdade de Odontologia, Universidade Federal de Minas Gerais, Belo Horizonte, Brazil; 5 Departamento de Análises Clínicas e Toxicológicas, Faculdade de Farmácia Universidade Federal de Minas Gerais, Belo Horizonte, Brazil; 6 Departamento de Patologia Geral, Instituto de Ciências Biológicas, Universidade Federal de Minas Gerais, Belo Horizonte, Brazil; 7 Departamento de Bioquímica e Imunologia, Instituto de Ciências Biológicas, Universidade Federal de Minas Gerais, Belo Horizonte, Brazil; 8 Departamento de Morfologia, Instituto de Ciências Biológicas, Universidade Federal de Minas Gerais, Belo Horizonte, Brazil; 9 Departamento de Física – Instituto de Ciências Exatas (ICEx), Universidade Federal de Minas Gerais, Belo Horizonte, Brazil; 10 Laboratory of Immunology Safety, State Research Center of Virology and Biotechnology “Vector”, Koltsovo, Russian Federation; 11 State Institute of Standardizing and Control by Name of Tarasevich, Moscow, Russian Federation; Texas Biomedical Research Institute, United States of America

## Abstract

There are few animal models of dengue infection, especially in immunocompetent mice. Here, we describe alterations found in adult immunocompetent mice inoculated with an adapted *Dengue virus* (DENV-3) strain. Infection of mice with the adapted DENV-3 caused inoculum-dependent lethality that was preceded by several hematological and biochemical changes and increased virus dissemination, features consistent with severe disease manifestation in humans. IFN-γ expression increased after DENV-3 infection of WT mice and this was preceded by increase in expression of IL-12 and IL-18. In DENV-3-inoculated IFN-γ^−/−^ mice, there was enhanced lethality, which was preceded by severe disease manifestation and virus replication. Lack of IFN-γ production was associated with diminished NO-synthase 2 (NOS2) expression and higher susceptibility of NOS2^−/−^ mice to DENV-3 infection. Therefore, mechanisms of protection to DENV-3 infection rely on IFN-γ-NOS2-NO-dependent control of viral replication and of disease severity, a pathway showed to be relevant for resistance to DENV infection in other experimental and clinical settings. Thus, the model of DENV-3 infection in immunocompetent mice described here represents a significant advance in animal models of severe dengue disease and may provide an important tool to the elucidation of immunopathogenesis of disease and of protective mechanisms associated with infection.

## Author Summary

Dengue is a mosquito-borne disease caused by one of four serotypes of *Dengue virus* (DENV-1-4). Dengue has escalated in geographic distribution and disease severity to become the most common arboviral infection of humans. There are no vaccines or specific therapies for dengue and the treatment is supportive. Immunopathogenesis of dengue disease is also poorly understood, in part, due to of the absence of proper animal models of infection. Here, we describe the phenotype of infection of immunocompetent mice with an adapted DENV-3 strain. Infection caused an inoculum-dependent lethality that was preceded by significant clinical, virological and biochemical changes resembling the severe manifestations of human infection. In addition, we demonstrate that IFN-γ production is essential for the host to deal with DENV-3 infection in a manner similar to that demonstrated previously for DENV-2. Hence, reduced IFN-γ production during DENV-3 infection was associated with diminished NOS2 expression and Nitric oxide production. Mice deficient for each of these molecules presented more severe disease manifestation and increased viral replication. Therefore, we describe a model of DENV-3 infection in immunocompetent mice that proves to be an interesting tool to study host–virus interactions and mechanisms mediating protection or those associated with severe disease manifestation.

## Introduction

Dengue viruses (DENV) are the most prevalent mosquito-borne RNA viruses worldwide, classified serologically into four antigenically distinct types (DENV-1–4). They are transmitted to humans by the mosquitoes *Aedes aegypti* and *Aedes albopictus* [1]–[3]. According to the World Health Organization (WHO) a total of 500,000 cases of dengue hemorrhagic fever (DHF) occur annually, and 20,000 deaths are estimated to happen every year [4]–[5]. The hallmark of severe dengue infection is a transient increase in vascular permeability, characterized by hemorrhagic manifestations, thrombocytopenia, and hemoconcentration, resulting in plasma leakage, which is believed to be immune mediated [4], [6]–[7]. Furthermore, deranged liver function is very common in patients with dengue infection and is generally manifested by the elevation of transaminase levels representing reactive hepatitis [8]–[10]. Treatment of dengue fever (DF) and of the severe forms of dengue infection is largely supportive [7], [11].

The pathogenesis of DENV infection remains poorly understood and involves a complex interplay of viral and host factors [1], [3], [6], [12]–[15]. The lack of a suitable animal model that emulate dengue disease, specially the severe forms (DHF/DSS), has hindered progress in many areas of dengue research, including pathogenesis, immunity, drug development and vaccine design and testing [7], [16]. Several studies in mice and humans have noted higher levels of viremia in severe dengue disease, which supports the assertion that increased viral replication is associated with more severe disease manifestation [17]–[21]. However, we and other groups have also demonstrated that inflammatory response, characterized by cytokine storm, also plays a fundamental role in dengue pathogenesis [7], [22]–[29]. Most of these studies were first characterized in a model of dengue infection using a mouse-adapted DENV-2 strain that mimics several clinical parameters seen in human disease, without affecting the CNS [22], [24]–[28]. In addition to characterizing mechanisms associated with pathogenesis, the DENV-2 model showed to be adequate to study pathways important for host resistance to infection. In this regard, we have recently demonstrated that IFN-γ production depends on IL-12 and IL-18 combined action and mediated host resistance to DENV-2 infection in a nitric oxide-dependent manner [30].

The development of animal models of all 4 DENV serotypes is extremely necessary and may help to determine: (i) whether different pathogenetic mechanisms operate in the different serotypes, (ii) the consequence of sequential infection and (iii) the efficacy of drugs and vaccine candidates [11], [31]. In this regard, in the present study we characterize a novel model of DENV-3 infection in immunocompetent adult mice, using the same strategy previously used for DENV-2 model. After inoculation of the adapted DENV-3 strain, we observed the occurrence of the major clinical manifestations of severe dengue infection, characterized by inoculum-dependent lethality that was preceded by significant clinical and biochemical alterations such as thrombocytopenia, hemoconcentration, plasma extravasation, liver damage with elevated AST/ALT levels in serum and massive cytokine production. Moreover, DENV-3 was detected in spleen and liver and viremia was detected from the fifth day of infection. There was also enhanced expression of NS3 in liver and NS1 concentration in plasma. The development of animal models for the four DENV serotypes will also allow to determine whether mechanisms of protection to infection are similar or not among the different serotypes. Hence, using this novel DENV-3 model, we demonstrate that the IFN-γ-induced Nitric Oxide production, found to be essential for host resistance to DENV-2 infection [30] plays a major role in host protection to DENV-3 infection. Mice deficient for IFN-γ and for NOS2 are markedly susceptible to DENV-3 infection, with elevated lethality rates, more severe disease and increased viral load after infection. Therefore, we describe a novel model of DENV-3 infection in immunocompetent mice that emulates many of the manifestations seen in human disease. The present model may provide an important tool to study host–virus interactions and mechanisms mediating protection or those associated with severe disease manifestation.

## Methods

### Ethics statement

This study was carried out in strict accordance with the Brazilian Government's ethical and animal experiments regulations. The experimental protocol was approved by the Committee on the Ethics of Animal Experiments of the Universidade Federal de Minas Gerais (CETEA/UFMG, Permit Protocol Number 113/09). All surgery was performed under ketamine/xylazine anesthesia and all efforts were made to minimize animal suffering.

### Animals

Mice deficient for IFN-γ and NOS-2 were obtained from The Jackson Laboratory and were bred and maintained at the Gnotobiology and Immunology Laboratory of Instituto de Ciências Biológicas. Mice deficient for IL-12p40 were kindly provided by Dr. J. Magran through Dr. L. V. Rizzo (Instituto de Ciências Biomédicas (ICB), University of São Paulo, São Paulo, Brazil) and were bred and maintained at the Gnotobiology and Immunology Laboratory of Instituto de Ciências Biológicas. Mice deficient for IL-18 [32] and IFNGR1 were kindly provided by Dr. F.Q. Cunha and were bred and maintained at the Gnotobiology and Immunology Laboratory of Instituto de Ciências Biológicas. All mice were on C57BL/6J genetic background (back-crossed at least 10 times) and wild-type control C57BL/6J (WT) mice were used. For experiments, 7–10 weeks old mice were kept under specific pathogen–free conditions, in filtered-cages with autoclaved food and water available *ad libitum*. Adult BALB/c mice (7–10 weeks) were also used. During DENV-3 virus adaptation process, newborn and BALB/c mice of different ages (1-4 weeks old) were maintained at the same conditions described above.

### Virus

A clinical isolate of Dengue virus type 3 (DENV-3), genotype I, was used (access number JN697379). All work with the infectious virus was performed in a BSL-2 facility of the Laboratório de Interação Microrganismo-Hospedeiro - ICB – UFMG. Dengue virus 3 (DENV-3) was adapted similarly as previously described [22]. Briefly, the virus had undergone two passages intracerebrally (ICR) in suckling mice. The brains of the moribund mice were harvested for preparing 10% (w/v) mice brain suspension in modified Eagle's medium (MEM). The death of the suckling mice was observed on day 5 after cerebral infection. After that, 10 sequential passages through BALB/c mice of different ages (1–4 weeks old) by intraperitoneal (i.p.) injection were performed. Two sequential passages were carried out for each age group of BALB/c mice. After each passage, the brains of the moribund mice were harvested for preparing 10% brain suspension and then used for the next passage. The last passage of DENV-3 obtained from the brain of 4-week-old BALB/c mice was used for five sequential passages in the brain of suckling mice ICR and was collected to produce stocks. Ten percent brain suspension served as virus stock and was stored at −70°C. In addition, virus stocks were produced from infected mosquito C6/36 cells, *in* vitro. To calculate virus titer, plaque assays were conducted in LLC-MK2 cells as described below. Viral titer of stock was 5,8×10^6^ PFU/mL of cell supernatant. Suspension from brain of non-infected mice was prepared in a similar way and was used as control in all experiments. In some experiments, the suspension of the adapted DENV-3 virus was UV-irradiated (exposure of virus stock for 15 min to a UV lamp producing irradiation predominantly at 365 nm) or heat inactivated (56°C for 1 h) before inoculation of mice.

### Experimental procedure

For infection experiments, the virus-containing brain suspensions were diluted in endotoxin-free PBS (3.2 mM Na_2_HPO_4_, 0.5 mM KH_2_PO_4_, 1.3 mM KCl, 135 mM NaCl) and injected i.p. into mice. For the evaluation of lethality, mice were inoculated i.p. and lethality rates evaluated every 12 h for 14 days. The various other parameters were evaluated at 3, 5 and 7 days or daily after i.p. inoculation of the virus. In all experiments using genetically deficient mice, relevant WT controls were performed in parallel. Non-infected animals were inoculated with brain suspension from non-infected suckling-mice diluted in a similar manner. In the experiments involving genetically deficient mice, the NI group represents the pooled results obtained from the analysis of deficient mice and WT non-infected mice. Results were pooled for ease presentation.

In some experiments IL-18 was neutralized by daily i.p. injection of 1mg/kg of recombinant human IL-18BP per animal (hIL-18 bp), starting 1 hour after DENV-3 inoculation and lasting until day 6 after virus inoculation. The dose was chosen based in a previous study of [33]. Control animals received the vehicle saline alone. The hIL-18 bp isoform was a kind gift of Dr. Amanda Proudfoot from Merck-Serono Pharmaceuticals (Geneve, Switzerland). In other experiments, mice were pretreated i.p with 100 µL anti-DENV-3 polyclonal antiserum or control serum, 60 min before inoculation of the adapted DENV-3. The anti-DENV serum utilized was kindly given by Dr. Ricardo Galler from Departamento de Bioquímica e Biologia Molecular do Instituto Oswaldo Cruz-Fiocruz, RJ, Brazil [34]. Serum was obtained from Rhesus monkeys *(macaca mullata)* inoculated subcutaneously on the anterior surface of the left forearm with 0,5 ml of the viral suspension containing 10^5^ PFU of the DENV-3 H87 (13 dpi) [34].

### Cell culture and in vitro infection studies

Murine bone marrow cells were isolated from femurs and were differentiated into myeloid DCs after culturing (change on days 3, 6, and 8) at 2×10^6^ cells/ml for 10 days in RPMI supplemented with 10% FCS and 4% J558L cell-conditioned medium as a source of GM-CSF as described [35]. DCs were plated in 96-well microculture plates (at 2×10^5^ cells/well in DMEM supplemented with 2 mM L-glutamine and 2×10^−5^ M 2-ME) and for infection, cells were incubated with 50 µL of the brain suspension containing DENV-3 at a MOI of 0,05 PFU/cell in the presence or not of IFN-γ (100 U/ml). Negative controls were stimulated with sterile brain suspensions submitted to the same procedures of the DENV-3 containing brain homogenate. For positive controls, cells were stimulated with TLR4 agonist LPS (*Escherichia coli*, serotype O111∶B4, Sigma-Aldrich, at 100 ng/ml). Cell supernatants were harvested after 72 h of stimulation for nitrite measurements by Griess reagents.

### Quantification of nitrite in cell supernatants

Cell-free culture medium was obtained by centrifugation and assayed for nitrite content, determined by the Griess method [36]. For this assay, 0.1 ml of culture medium or serum was mixed with 0.1 ml of Griess reagent in a multiwell plate, and the absorbance at 550 nm read 10 min later. The NO_2_^−^ concentration was determined by reference to a NaNO_2_ standard curve (1 to 200 µM).

### Estimation of nitric oxide production ex vivo

Diaminofluorescein diacetate (DAF-2DA), a non-fluorescent cell permeable dye, was used. For NO estimation, esplenocytes of non-infected and DENV-3- infected mice were isolated (10^6^ cells/well) and incubated with 10 µM, DAF-2DA for 30 min at 37°C and fluorescence was determined in a fluorometer (Synergy 2, BIOTEK, USA) at excitation wave length 488 nm and measuring emission at 515 nm. Data were expressed as mean ± SEM of fold increase of fluorescence over stained-esplenocytes of NI mice.

### Titration of virus

Mice were assayed for viral titers in blood, brain, spleen and liver. Blood samples (50 µL) were collected in heparinized tubes, diluted in 450 µL of endotoxin-free PBS (3.2 mM Na_2_HPO_4_, 0.5 mM KH_2_PO_4_, 1.3 mM KCl, 135 mM NaCl) and stored at −70°C. For virus recovery in brain, spleen and liver, the organs were collected aseptically in different time points and stored at −70°C until assayed for DENV-3 virus. Tissue samples were weighed, grounded by using a pestle and mortar and prepared as 10% (w/v) homogenates in minimal essential medium (MEM) without fetal bovine serum (FBS). Viral load in supernatants of tissue homogenates and blood samples were assessed by direct plaque assay using LLC-MK2 cells as described [24], [27]. In Brief, LLC-MK2 cells were seed in 6 well plates and grown to confluence. 24 hours later, cell layers were incubated with serially diluted 0.4 mL of virus samples for 1 and half hour and overlaid with 1,5% methylcellulose +199 medium, 3% FBS. Plates were incubated for 7 days at 37°C, fixed in 10% formaldehyde, and stained with 1% crystal violet in water for 30 min. Plaques were counted by eye. The results were measured as plaque forming units (PFU) per gram of tissue weight or per mL of blood. The limit of detection of the assay was 100 PFU/g of tissue, or per mL.

### In vitro plaque purification

Plaque purification was performed as previously described [37]. Briefly, LLC-MK2 cells were seeded in a 6-wells plate and grown to 80% confluence. Then, cells were infected with different inoculums of the brain adapted DENV-3, overlaid with agarose 0,5% prepared in DMEM 5% FBS. Culture was incubated at 37°C, 5% CO2 for 7 days and single plaques were picked for expansion in LLC-MK2 cells. Nine clones were obtained and were titrated in LLC-MK2 cells for further *in vivo* evaluation.

### Evaluation of blood parameters

Blood was obtained from the cava vein in heparin-containing syringes at the indicated times under ketamin and xylazine anesthesia (150 mg/Kg and 10 mg/Kg, respectively) .The final concentration of heparin was 50 u/ml. Platelets were counted in a Neubauer chamber. Briefly, 10 ul of solution (amonium oxalate 1% and blood in a dilution of 1∶100) were placed in the chamber and platelets were visualized in a Nikon XP-1000 microscope, magnification of 400×, using phase contrast. Results are presented as number of platelets per µl of blood. For the determination of the hematocrit, a sample of blood was collected into heparinized capillary tubes (Perfecta) and centrifuged for 10 min in a Hematocrit centrifuge (Fanem, São Paulo, Brazil).

### Detection of Dengue Virus NS1 Antigen

*Dengue virus* NS1 antigen was measured in individual serum samples (1∶3 dillution), using a commercially ELISA available kit (BIO-RAD Platelia ™ Dengue NS1 AG). The optical density reading obtained with a spectrophotometer set at 450 nm was proportional to the amount of NS1 antigen present in the sample. Results are expressed as absorbance at 450 nm.

### Measurement of cytokines/chemokine concentrations

The concentration of cytokines (TNF-α, IFN-γ, IL-6, IL-12p40 and IL-18) in serum or tissue samples was measured using commercially available antibodies and according to the procedures supplied by the manufacturer (R&D Systems, Minneapolis, except for IL-18, manufactured by BD Pharmingen). Results are expressed as pg/mL or pg/100 mg of tissue. The detection limit of the ELISA assays was in the range of 4–8 pg/ml.

### Evaluation of changes in vascular permeability

The extravasation of Evans blue dye into the tissue was used as an index of increased vascular permeability, as previously described [38]. Briefly, Evans blue (20 mg kg^−1^) was administered i.v. (1 ml kg^−1^) via an eye vein 30 min prior to mice sacrifice. After that, one lobe of liver and the left lung were cut and allowed to dry in a Petri dish for 24 h at 37.^C^. The right ventricle was flushed with 10 ml of phosphate-buffered saline (PBS) to wash the intravascular Evans blue in the lungs. The left lung was then excised and used for Evans blue extraction. The dry weight of the tissue was calculated and Evans blue extracted using 1 ml of formamide (24 h at room temperature). The amount of Evans blue in the tissue was obtained by comparing the extracted absorbance with that of a standard Evans blue curve read at 620 nm in an ELISA plate reader. Results are presented as the amount of Evans blue per mg per 100 mg of tissue.

### Transaminase activity

The transaminases Aspartate aminotransferase (AST) and Alanine aminotransferase (ALT) activity were measured in individual serum samples, using a commercially colorimetric available kit (Bioclin, Quibasa, Belo Horizonte, Brazil). Results are expressed as the mean mean ± SEM of transaminase concentration in U/dL of plasma.

### Body weight and hemodynamic measurements

Body weight (BW) and systolic blood pressure (SBP) were measured in uninfected and infected mice on days 0,3,4,5,6 and 7 after infection. All animals were habituated to the blood pressure measurement device for 7 days. SBP was determined with tail-cuff plethysmography method in unanesthetized mice, as previously described [39]. All data are expressed as mean ± SEM. Changes in SBP from baseline are expressed as absolute values as well as areas under the BP curves.

### Hypernociception assessment by a modified electronic pressure-meter test for mice

Hypernociception was assessed as described by Sachs et al, 2010 [40]. Briefly, mice were placed in acrylic cages with a wire grid floor 15–30 min before testing for environmental adaptation. In these experiments, an electronic pressure-meter was used. It consists of a hand-held force transducer fitted with a polypropylene tip (INSIGTH Instruments, Ribeirão Preto, SP, Brazil) [41]. A standard large tip (0.5 mm^2^) was applied in the hind paw of the DENV-3 infected mice or it respective controls and an increasing perpendicular force was applied to the central area of the plantar surface of the hind paw to induce the flexion of the knee joint, followed by paw withdraw. After the flexion-elicited withdrawal threshold, the intensity of the pressure was automatically recorded. The value for the response was obtained by averaging three measurements. Animals were tested daily after inoculation. Results are expressed as Δ withdrawal threshold (g) calculated by subtracting zero-time mean measurements from the time interval mean measurements.

### Conventional PCR and real time PCR

For typing of adapted-DENV-3 virus, RNA was extracted with QIAMP viral RNA kit (Qiagen, Hilden, Germany) from the adapted-DENV-3 after different passages in C6/36 mosquito cells and of clone 4 obtained from the adapted DENV-3. The non-adapted DENV-3 was also used as control. First-strand cDNA synthesis for subsequent PCR assays was performed with approximately 400 ng of total RNA and random primer C118A (PROMEGA, Madison). A PCR assays was performed with specific primer combinations D1/TS3 (DENV-3), previously described by [42]. PCR products were run on a 1.5% agarose gel stained with ethidium bromide.

For evaluation of NOS2 mRNA expression, spleens were removed 3, 5 and 7 days after DENV-3 inoculation into mice. Total RNA was isolated from tissues by using a QIAGen RNEasy RNA Isolation Kit. The RNA obtained was resuspended in diethyl pyrocarbonate treated water and stocked at −70°C until use. Real-time RT-PCR was performed on a StepOne sequence-detection system (Applied Biosystems) by using SYBR Green PCR Master Mix (Applied Biosystems) after a reverse transcription reaction of 2 µg of RNA by using M-MLV reverse transcriptase (Promega). The relative level of gene expression was determined by the comparative threshold cycle method as described by the manufacturer, whereby data for each sample were normalized by 18S ribosomal RNA and expressed as a fold change compared with non-infected controls or medium cultivated cells. The following primer pairs were used: *18S ribosomal RNA*, 5′-CGTTCCACCAACTAAGAACG-3′ (forward) and 5′-CTCAACACGGGAAACCTC AC-3′ (reverse); and *nos2*, 5′- AGCACTTTGGGTGACCACCAGGA-3′ (forward) and 5′- AGCTAAGTATTAGAGCGGCGGCA -3′ (reverse).

### FACS analysis

Spleen cells were evaluated *ex vivo* for extracellular molecular expression patterns and for intracellular cytokine expression patterns. Briefly, spleens were removed from infected mice on day 7 after infection and cells were isolated, and immediately stained for surface markers, fixed with 2% formaldehyde and then permeabilized with a solution of saponin and stained for 30 min at room temperature, using anti-IFN-γ monoclonal antibodies directly conjugated with FITC. Preparations were then analyzed using a FACScan (Becton Dickinson), gating on a total lymphocyte/monocyte population. The antibodies used for the staining were rat immunoglobulin control(s), anti-CD4-PE, anti-CD8-PE, anti-NK1.1-PE, anti-CD3-biotin and anti-IFN-γ-FITC (all from Biolegend Inc). For detection of CD3 staining, cells were incubated with streptavidin conjugated to PE-Cy5 fluorochrome (Serotec Inc) for 30 min at 4°C before fixing. Spleen cells were analyzed for their intracellular cytokine expression patterns and frequencies using the software Flow Jo 7.2 (Tree Star Inc). The frequency of positive cells was analyzed using a gate that included lymphocytes, large blast lymphocytes and monocytes/macrophages. Limits for the quadrant markers were always set based on negative populations and isotype controls.

### Histopathological analysis

Liver samples from adult euthanized mice were obtained at the indicated time points. Afterwards, they were immediately fixed in 10% buffered formalin for 24 hours and embedded in paraffin. Tissue sections (4 µm thicknesses) were stained with hematoxylin and eosin (H&E) and evaluated under a microscope Axioskop 40 (Carl Zeiss, Göttingen, Germany) adapted to a digital camera (PowerShot A620, Canon, Tokyo, Japan). Histopathology score was performed according to a set of custom designed criteria modified from [43] evaluating hepatocyte swelling, degeneration, necrosis and hemorrhage, added to a five-points score (0, absent; 1, minimal; 2, slight; 3, moderate; 4, marked; and 5, severe) in each analysis. For easy interpretation, the overall score was taken into account and all the parameters totalized 20 points. A total of two sections for each animal were examined and results were plotted as the media of damage values in each mouse.

### Immunohistochemistry analysis

For immunohistochemistry, sections were treated with 3% H_2_O_2_ diluted in Tris-buffered saline (TBS) (pH 7.4) for 30 minutes. For antigen retrieval, tissue sections were immersed in citrate buffer (pH 6.0) for 20 minutes at 95°C. For NOS2 detection the slides were then incubated with the rabbit polyclonal anti-NOS2 (N-20, sc-651, Santa Cruz Biotechnology, Santa Cruz, CA) diluted 1∶100; at 4°C overnight in a humidified chamber. For detection and quantification of DENV-3 infected cells an anti-DENV NS3 MAb E1D8 or an isotype control was used in a dilution of 1∶350 for liver and 1∶100 for brain; at 4°C overnight in a humidified chamber. After incubation, tissue sections were washed with TBS and treated with a labeled streptavidin-biotin kit EnVision® + Dual Link System-HRP (Dako). Sections were then rinsed in PBS with 3,3′-diaminobenzidine tetrahydrochloride (K3468, Dako) for 5 minutes and stained with Mayer's hematoxylin. For quantification of NOS-2^+^ cells or NS3^+^ cells, cells counts were performed in 10 alternate microscopic high-power fields (×400) for each sample (4–5 mice per group). It was counted the number of positive hepatocytes, kupffer cells and inflammatory cells in each field. Areas of necrosis and hemorrhage were excluded from the analysis. The distribution of NS3 was assessed throughout the brain on at least two different brain coronal sections.

### Intravital confocal microscopy

Liver intravital microscopy was performed as previously described [44]. Briefly, mice were anesthetized as describe previously. Mice were placed in a right lateral position on an adjustable microscope stage. A lateral abdominal incision along the costal margin to the midaxillary line was made to exteriorize the liver, and all exposed tissues were moistened with saline-soaked gauze to prevent dehydration. The liver was placed on a stage for an upright microscope and the liver surface was then covered with a coverslip to hold the organ in position. The liver was visualized using intravital multiphoton and confocal microscopy system based on a modified Olympus confocal microscope (FV300) in an up- right configuration (BX51 Microscope). The images presented were obtained using the confocal laser at 488 nm using a 10/0.30 UplanFLN objective. Cells were fluorescently labeled by rhodamine 6G (0,05%; i.v.) to assess hepatocyte size by measuring the longest cell axis of 20–30 cells/field (Image J, NIH, USA). Sinusoids were labeled by i.v. injection of phycoerythrin-anti PECAM-1 (0,5 µg/mice ; PE anti-CD31, clone 390 ; Ebioscience, USA) and the percentage of perfused sinusoids was assessed by digital quantification of the area fraction stained by the antibody (Image J, NIH, USA).

### Statistical analysis

Results are shown as means ± S.E.M. Percent inhibition was calculated by subtracting the background values obtained in non-infected animals. Differences were compared by using analysis of variance (ANOVA) followed by Student-Newman-Keuls post-hoc analysis. Differences between lethality curves were calculated using Log rank test (Graph Prism Software 4.0). Changes in SBP from baseline are expressed as absolute values as well as areas under the BP curves. Results with a P<0.05 were considered significant.

## Results

### Disease parameters in immunocompetent mice infected with an adapted strain of DENV-3

**Figure 1:**
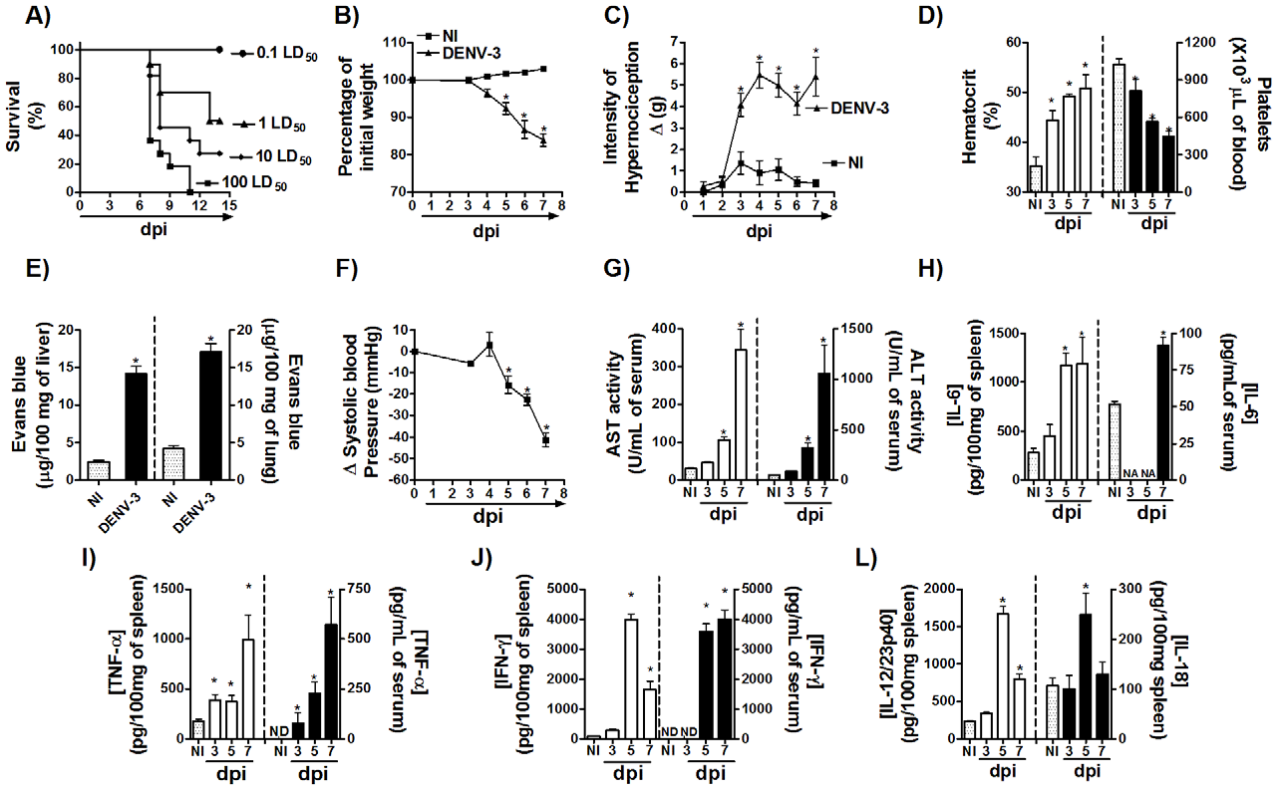
Disease parameters in C57BL/6 mice infected with an adapted strain of DENV-3. (A) WT mice (*n* = 6 mice per group) were inoculated with different inoculums of adapted-DENV-3 (i.p) and lethality was evaluated every 12 hours for 14 days. Results are expressed as % of survival. In Figs (B–L) WT mice (n = 6 per group) were inoculated with 10LD_50_ (1000 PFU) of DENV-3 (i.p) and in the third, fifth or in the seventh day of infection mice were culled and blood and tissues were collected for the following analysis: (B) Change in body weight was expressed as percentage of initial weight loss. (C) Mechanical hypernociception was assessed daily. Results are shown as the difference between the force (g) necessary to induce dorsal flexion of tibio-tarsal joint, followed by paw withdraw, before and after DENV-3 inoculation. In (D), hematocrit was expressed as % volume occupied by red blood cells (left panel) and the number of platelets was shown as platelets ×10^3^/µl of blood (right panel). (E) Changes in vascular permeability in the liver and lungs are shown as µg Evans blue per 100 mg of tissue (left and right panels, respectively). (F) Shows changes in Systolic blood pressure from baseline until day 7 after infection expressed as Δ of blood pressure in mmHg. (G) AST (left panel) and ALT (right panel) activity determination in plasma of control and DENV-3-infected mice was shown as U/dL of plasma. (H–L) Concentrations of IL-6, TNF-α, IFN-γ IL-12/23p40 and IL-18, quantified by ELISA. Results are shown as pg per mL (serum) or pg per 100 mg (tissue). All results are expressed as mean ± SEM and are representative of at least two experiments. * for P<0.05 when compared to control uninfected mice. 10 LD_50_ corresponds to 1000 PFU of adapted-DENV-3. ND – not detectable. NA – not assessed. NI- Not-infected. dpi – days post-infection.

**Figure 2:**
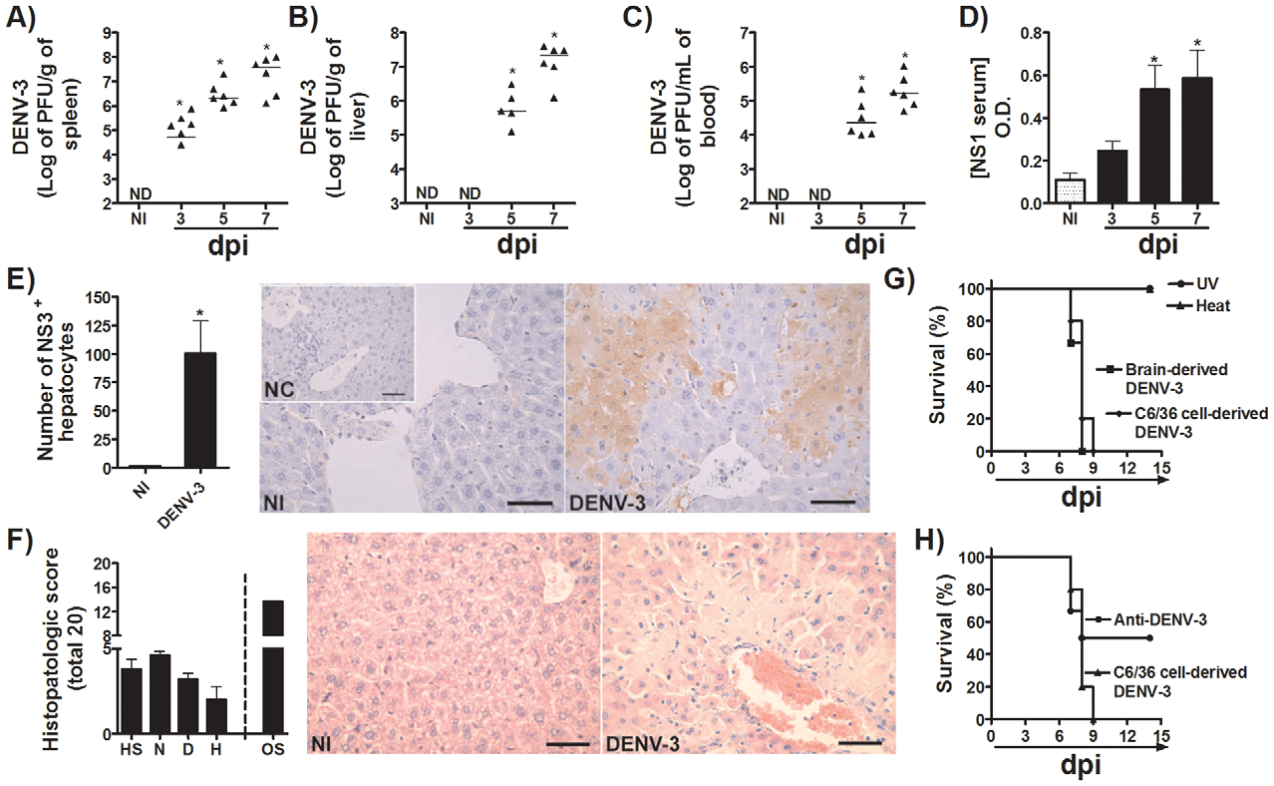
Characterization of virologic and histopathological parameters in C57BL/6j mice upon adapted-DENV-3 infection. (A–D) C57BL/6j mice (n = 6 per group) were inoculated with 10LD_50_ (1000 PFU) of DENV-3 (i.p) and in the third, fifth or in the seventh day of infection, mice were culled and blood and tissues were collected for the following analysis: (A–C) Viral loads were recovered from the spleen, liver and blood, respectively. Results are shown as the log of PFU per g of tissue or per mL of blood. (D) Shows virus NS1 antigen serum levels by ELISA and expressed as O.D. (E–F) C57BL/6j mice (n = 6 per group) were inoculated with 10LD_50_ (1000 PFU) of DENV-3 (i.p) and in the seventh day of infection mice were culled and liver collected for the following analyses: (E) Liver was collected, formalin-fixed and processed into paraffin sections. Serial sections from each tissue were stained with anti-DV NS3 antibody E1D8 (NS3) or an isotype control mouse IgG2a, and multiple sections of each tissue type were thoroughly examined for staining. Positive staining for NS3 is brown while hematoxylin counterstain is blue. (F) shows semi-quantitative analysis of hepatic damage and Hematoxylin & Eosin staining of liver sections of control and DENV-3-infected mice, seven days after infection (Scale Bar - 400 µm). The images presented are representative of an animal on the seventh day of infection. In (G) Viral inoculum (10LD_50_ or 1000 PFU) was heat inactivated (Heat, 56°C, 60 min) or treated with UV light (UV, 15 min) before inoculation in C57BL/6j mice. Lethality was evaluated every 12 hours for 14 days. (H) WT mice (*n* = 6 mice per group) were pretreated i.p with 100 µL of Anti-DENV-3 antiserum or control serum (pre-immune serum) before inoculation of 10LD_50_ (1000 PFU) of adapted-DENV-3 (i.p). Lethality was evaluated every 12 hours for 14 days. Results are expressed as % of survival. Results are expressed as mean ± SEM (except for A–C, expressed as median) and are representative of at least two experiments. * for P<0.05 when compared to control uninfected mice. 10 LD_50_ corresponds to 1000 PFU of adapted-DENV-3. ND- not detected. NI- not-infected. dpi- days post-infection. NC – Negative control. HS – hepatocyte swelling. N – necrosis. D – degeneration. H – hemorrhage. OS – Overall Score.

Infection of adult C57BL/6j (Figure 1A) or BALB/c (Figure S1A) mice with an adapted strain of DENV-3 induced an inoculum-dependent lethality that was usually observed around the 7^th^ or 6^th^ days after inoculation of DENV-3, respectively. Next, we performed series of experiments to characterize the disease caused by the adapted DENV-3 in both mice strains. In all experiments, control mice were inoculated with brain suspension which caused no clinical or biochemical alterations in comparison with non-inoculated mice (data not shown). Infection kinetic studies were carried out with an inoculum of 10LD_50_ and 1LD_50_ for C57BL/6j and BALB/c mice strains, respectively. Inoculi were equivalent to 1000 and 100 PFU, respectively, as verified by plaque assay in LLC-MK2 cells. Experiments were conducted till day 7, the peak of infection, as there was significant lethality in WT mice after this period (Figures 1A and S1A). Lethality of both strains of adult mice infected with DENV-3 was preceded by significant changes in clinical and biochemical parameters as shown in Figures 1, 2 and S1. There was marked weight loss, beginning at day 4 after infection, reaching about 20% on day 7 after DENV-3 inoculation (Figure 1B and S1B). There was also significant hypernociception in response to mechanical stimulation, an index of pain in experimental animals, lasting from day 3 until day 7 post-infection (Figure 1C and S1C). In addition, DENV-3 infection induced significant hematological alterations. Thrombocytopenia was observed as early as 3 days after infection and platelets counts were around 50% of normal at day 7 (Figure 1D and S1D, right panels). The hematocrit, a marker of hemoconcentration, was elevated from day 3 and increased to greater than 50% by day 7 (Fig. 1D and S1D, left panel). In addition to hemoconcentration, there was marked plasma extravasation in target organs, as assessed by increase in concentration of Evans blue dye in liver and lungs, respectively (Fig. 1E). These findings were accompanied by changes in hemodynamic parameters, showed by reduction in systolic blood pressure, more sharply on day 7 p.i. Hence, at this time point, there was a striking 40 mmHg fall in systolic blood pressure in DENV-3-infected mice (Figure 1F). The concentration of liver enzymes in serum (Aspartate aminotransferase [AST] and Alanine aminotranferease [ALT]) were elevated after DENV-3 infection. There was an increase of AST and ALT of approximately 10 and 30 times, respectively, at day 7 after infection in both strains (Figure 1G and S1E). Evaluation of the liver microvasculature by intravital confocal microscopy revealed a significant increase in hepatocyte diameter at day 7 after DENV-3 inoculation as compared to non-infected mice (Figure S2A). There was a decrease of sinusoidal perfusion which paralleled the increase in hepatocyte diameter (Figure S2B). The levels of IL-6, TNF-α, IFN-γ, IL-12/23p40 and IL-18 were evaluated in spleen or serum of infected mice. Overall, there was a good correlation between levels of cytokines in serum and spleen and the severity of disease (Figure 1H-L). IL-6 levels were elevated at 5 and 7 dpi in spleen and 7 dpi in serum after DENV-3 inoculation (Figure 1H). Levels of TNF-α rose rapidly from day 3 in spleen and serum of infected mice, peaking on day 7 (Figure 1I). Nevertheless, IFN-γ peaked on day 5, and still remained elevated at day 7 in spleen and serum of DENV-3 infected mice as compared to NI group (Figure 1J). There were detectable levels of both IL-12/23p40 (Figure 1L, left panel) and IL-18 (Figure 1L, right panel) cytokines in the spleen of WT mice already on day 5 of infection. IL-18 levels reduced to basal values at the 7^th^ day of infection, while IL-12/23p40 remained above background levels at this time point (Figure 1L right and left panels, respectively).

Of note, neither limb paralysis nor any other sign of CNS inflammation (eg. levels of TNF-α and IL-6 in brain) were noticed after peripheral inoculation of DENV-3 into adult mice (data not shown). Therefore, in summary, we show that immunocompetent adult mice infected systemically with an adapted strain of DENV-3 virus presented several clinical and pathological systemic features that resemble severe dengue disease in humans.

### Characterization of virological and histopathological parameters in immunocompetent mice infected with DENV-3

After inoculation, the virus was detected from day 3 in the spleen (Figure 2A and S3A), from day 5 in liver (Figure 2B and S3B), and there was significant viremia from day 5 p.i. (Figure 2C and S3C) in C57BL/6j and BALB/c mice, respectively. Viral load escalated further at day 7 in all tissue aforementioned (Figure 2A–C and S3A–C). In addition to high viremia, serum levels of dengue virus NS1 antigen was increased on days 5 and 7 after DENV-3 infection (Figure 2D). The presence of DENV-3 in the liver tissue of infected mice was also investigated by immunohistochemistry assay using an anti-dengue NS3 antibody. As expected, negative controls did not present any positive reaction (Figure 2E). On the other hand, we found NS3-positive staining in liver of DENV-3 inoculated mice (Figure 2E), demonstrating active viral replication of the virus in this target organ. The histoquantitative analyses revealed elevated number of cells expressing virus antigens on day 7 after DENV-3 inoculation. Of the total number of NS3-positive cells, 84% were hepatocytes, whereas there were also 8% kupffer cells and 7% inflammatory cells, as assessed morphologically. These results suggested that DENV-3 replicates in such cells, mainly in hepatocytes, which are present in the same areas of tissue damage (Figure 2E). Corroborating this data, marked hepatic injury was found in DENV-3 infected mice at day 7 p.i. (Figure 2F). Histopathological analyses revealed intense multifocal to coalescing areas of hemorrhagic necrosis (Figure 2F). Overall, all infected mice exhibited inflammatory infiltrates composed of neutrophils, macrophages and lymphocytes around blood vessels (portal and central veins) and scattered throughout the parenchyma. Moderate to intense hepatocyte swelling and degeneration were also detected. The total score in DENV-3 infected group was 13.6±4.2 points, in a total of 20 points, demonstrating a significant degree of liver injury, possible directly associated with the higher viral replication in this organ.

DENV-3 obtained from C6/36 cell culture supernatant induced disease that was very similar to the disease induced by viral stocks prepared from brain suspension (Figure 2G-H and S3D). UV irradiation or heat inactivation of the virus prevented lethality and any form of clinical manifestation of C57BL/6j mice *in vivo* (Figure 2G and data not shown). Figure S4A demonstrates typing of adapted-DENV-3 after several passages in mosquito C6/36 cells. Moreover, treatment of C57BL/6j mice with an anti-DENV-3 polyclonal antiserum obtained from DENV-3-infected monkeys reduced the mortality rate of adapted-DENV-3 infected mice to approximately 50% (Figure 2H).

Plaque purification technique was performed to isolate DENV-3 clones and test the capacity of these clones to induce severe disease. We obtained nine DENV-3 clones that were expanded in LLC-MK2 cells and tested *in vivo*. These clones were designated clones 1 to 9. BALB/c mice that were infected by the DENV-3 clones 1, 2 or 5–9 did not present any form of clinical manifestation and therefore 100% survived to inoculation (Figure S3D). However approximately 25% of mice infected with DENV-3 clone 3 progressed to death (Figure S3D). In addition, all mice infected with DENV-3 clone 4, BALB/c (Figure S3D) or C57BL/6j (Figure S5A) strains, presented severe clinical manifestation of disease showed by enhanced mortality rate (Figure S3D and S5A), viremia, thrombocytopenia and hemoconcentration (Figure S5B–D), similarly to mice infected with adapted-DENV-3 grown in C6/36 or brain-derived DENV-3 adapted virus. Figure S4B demonstrates typing of adapted DENV-3 (clone-4) after passage in LLCMK-2 cells. In addition, we have performed subsequent rounds of plaque purification of the adapted Clone 4 of DENV-3 and we have found that injection of a clone from the clone in adult mice induced disease that was similar to the disease seen in mice infected with the original clone (hematocrit: NI: 38.8±0.9%; DENV-3 (7^th^ dpi): 45±1.3%, p = 0.014; platelet counts: NI: 891±22×10^3^/µL of blood; DENV-3 (7^th^ dpi): 570±31×10^3^/µL of blood, p<0.001)

To evaluate the presence of virus in the CNS, we measured number of DENV-3 plaques after systemic or CNS inoculation in adult and weaning mice. Systemic injection of the virus in adult mice resulted in detection of the virus in spleen (Figure 2A and S6A) but no detection of virus in the brain (Figure S6B). In contrast, systemic inoculation of the virus in weaning mice resulted in significant detection of the virus in the brain (Figure S6B). However, the viral load in the CNS was much higher when the virus was inoculated directly into the brain (Figure S6B). Immunohistochemistry analysis concurred with the findings above and revealed that NS3 staining was only detect in newborn mice and specially after direct injection into the brain (Figure S6C). These data corroborate with the absence of neurologic symptoms during disease, as well as with the lack of cytokine production in brain tissue, as discussed above. Therefore, the adapted DENV3 strain still maintains its neurotropism in newborn mice which is lost as mice ages and probably correlates with the development of the blood brain barrier.

### Production of IFN-γ is required for host resistance to adapted-DENV-3 primary infection

**Figure 3:**
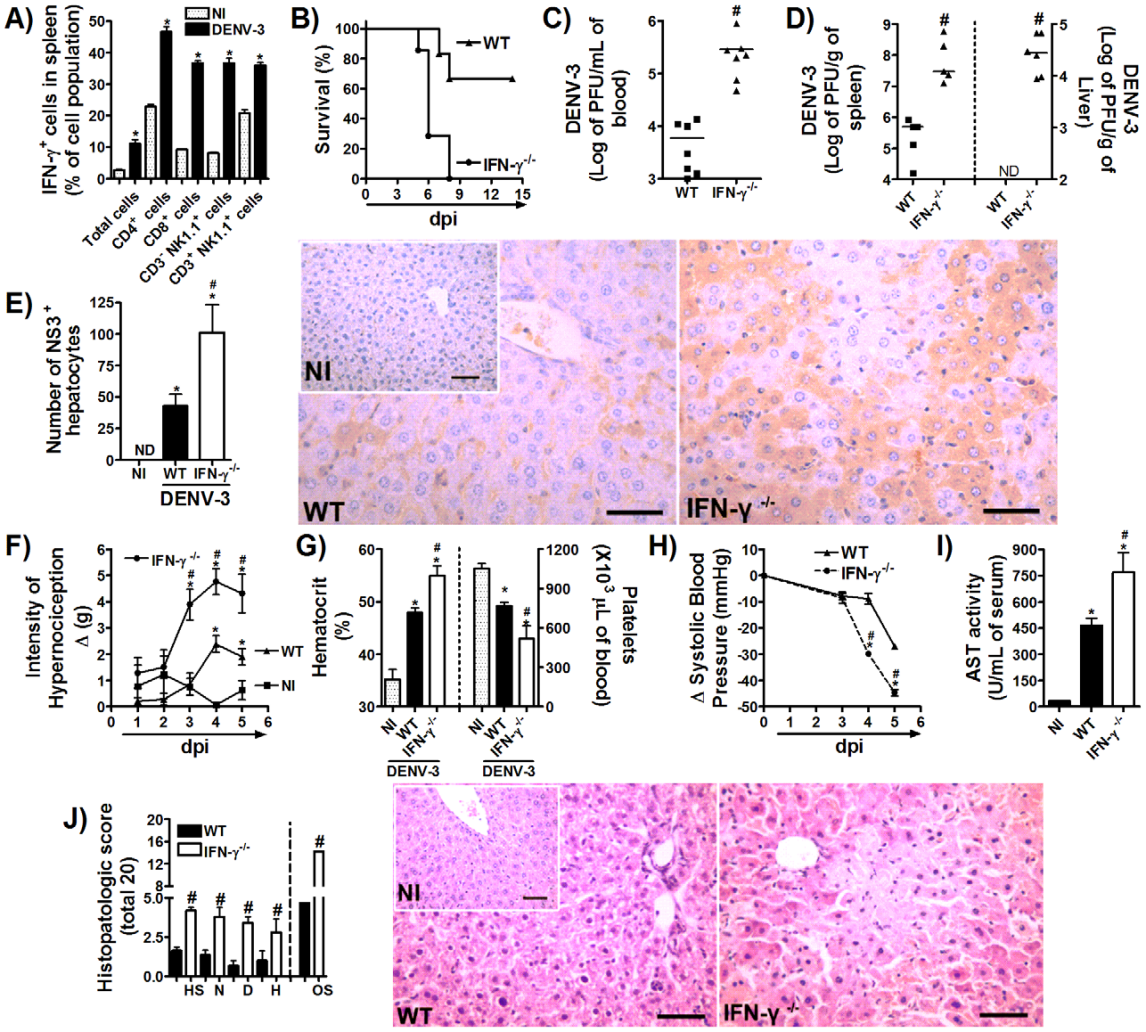
IFN-γ production is required for host resistance to adapted-DENV-3 primary infection. (A) WT mice (*n* = 4 mice per group) were inoculated with 10LD_50_ (1000 PFU) of DENV-3 (i.p) and seven days later, mice were culled, and splenic cells isolated for assaying IFN-γ production by cellular staining with labeled antibodies and FACS analysis. Results are expressed as % of IFN-γ-positive cells in each population. (B) WT and IFN-γ^−/−^ mice (n = 8 per group) were inoculated with 1LD_50_ (100 PFU) of DENV-3 (i.p) and lethality was evaluated every 12 hours during 14 days. Results are expressed as % of survival. In (C–J), WT and IFN-γ^−/−^ mice (n = 6 per group) were inoculated with 1LD_50_ (100 PFU) of DENV-3 (i.p) and in the fifth day of infection mice were culled and blood and tissues were collected for the following analysis: (C–D) Viral loads were recovered from the blood (C), spleen and liver (D, left and right panels), respectively. Results are shown as the log of PFU per mL of blood or per g of tissue. (E) Serial sections from each liver were stained with anti-DV NS3 antibody E1D8 (NS3) or an isotype control mouse IgG2a (IgG2a data not shown), and multiple sections of each tissue type were thoroughly examined for staining. Positive staining for NS3 is brown while hematoxylin counterstain is blue. Results are expressed as number of NS3-positive hepatocytes. (F) Mechanical hypernociception was assessed daily. Results are shown as the difference between the force (g) necessary to induce dorsal flexion of tibio-tarsal joint, followed by paw withdraw, before and after DENV-3 inoculation. In (G), hematocrit was shown as % volume occupied by red blood cells (left panel) and the number of platelets was shown as platelets ×10^3^/µl of blood (right panel). (H) Changes in Systolic blood pressure from baseline until day 5 after infection expressed as Δ of blood pressure in mmHg. In (I), AST activity determination in plasma, shown as U/dL of plasma. (J) shows semi-quantitative analysis of hepatic damage (histopathological analysis performed as modified from Paes et al, 2009) and Hematoxylin & Eosin staining of liver sections of control and WT and IFN-γ^−/−^ DENV-3-infected mice, five days after infection. Scale bars - 400 µm. The images presented are representative of an animal on the fifth day of infection. All results are expressed as mean ± SEM (except for C–D, expressed as median) and are representative of at least two experiments. * for P<0.05 when compared to control uninfected mice. # fo P,0.05 when compared to WT infected mice. 10 LD_50_ corresponds to 1000 PFU of adapted-DENV-3. 1LD_50_ corresponds to 100 PFU of adapted-DENV-3. ND – not detectable. NI- Not-infected. dpi – days post-infection. HS – hepatocyte swelling. N – necrosis. D – degeneration. H – hemorrhage. OS – Overall Score.

As shown in Figure 1J, during the time course of DENV-3 infection, there was an increase in levels of IFN-γ in serum and spleen from the 5^th^ day of infection that was maintained at the 7^th^ day p.i. (Figure 1J, right and left panels, respectively). In addition, IFN-γ levels in the liver of adapted-DENV-3 mice were also increased from day 5, reaching higher values on day 7 (NI = 36±11 pg/100 mg of tissue; 3 d = 64±6 pg/100 mg of tissue; 5 d = 109±11 pg/100 mg of tissue; 7 d = 216±31 pg/100 mg of tissue; n = 6, p<0.05). FACS analysis of esplenocytes isolated from DENV-3 infected mice revealed IFN-γ staining in about 12% of total cells on 7^th^ day after inoculation (Figure 3A). There was an increase in expression of IFN-γ in CD4^+^ T cells, CD8^+^ T cells, CD3^−^NK1.1^+^ NK cells, and CD3^+^NK1.1^+^ NKT cells. Significantly, over 46% of CD4^+^ T cells, 36% of CD8^+^ T cells and 36% of CD3^−^NK1.1^+^ NK cells and 35% of CD3^+^NK1.1^+^ NKT cells were IFN-γ^+^ at this period in comparison with cells of non-infected mice (Figure 3A).

To investigate in vivo the role played by IFN-γ during DENV-3 infection, wild type (WT) and IFN-γ deficient (IFN-γ^−/−^) mice were inoculated with 1LD_50_ of adapted DENV-3 and mortality rate and disease parameters were evaluated. After infection, 100% of IFN-γ^−/−^ mice were dead before day 9 of infection, while less than 30% of WT mice had succumbed to infection after 14 days of inoculation of DENV-3 (Figure 3B). In fact, approximately 75% of IFN-γ^−/−^ mice were already dead at day 6 after infection (Figure 3B), which led us to perform the subsequent analysis on day 5 after infection. The early lethality of IFN-γ^−/−^ mice was associated with increased DENV-3 replication. As early as the 3^rd^ day of DENV-3 inoculation, viremia in IFN-γ^−/−^ mice was detectable (WT: not detectable; IFN-γ^−/−^: 2.9×10^3^ PFU/mL of blood, n = 4, p = 0,03). Viremia was almost 2log higher in IFN-γ^−/−^ mice in comparison to WT mice at 5 days after infection (Figure 3C). Indeed, at this time point, there was marked increase of viral load in spleen (Figure 3D, left panel) and liver (Figure 3D, right panel) of infected IFN-γ^−/−^ mice. Moreover, NS3^+^ staining in liver was strikingly higher in infected IFN-γ^−/−^ mice when compared with their WT littermates (Figures 3E). Again, the hepatocytes were the predominant cell stained for NS3 protein, representing almost 90% of positive cells. Of interest, the virus could not be detected in the brain of WT and IFN-γ^−/−^ infected mice (data not shown).

In addition to greater lethality rates and to enhanced viral replication, IFN-γ^−/−^ mice presented more severe manifestation of disease after infection (Figure 3F–J). Hypernociception initiated earlier and remained increased at all time points evaluated (Figure 3F). IFN-γ^−/−^ mice also had more marked thrombocytopenia (Figure 3G, right panel), significant greater increase in hematocrit values (Figure 3G, left panel), and more drastic reduction in systolic blood pressure (Figure 3H) than infected WT mice.

IFN-γ^−/−^ mice showed greater liver injury after DENV-3 infection, as demonstrated by increased AST activity in plasma (Figure 3I). There were marked histopathological alterations in liver of IFN-γ^−/−^ mice which were more intense than those of WT mice (Figure 3J) but similar to those described in WT mice infected with a higher inoculum (10LD_50_) (Figure 2F). It is noteworthy that the inoculum used (1LD_50_) was capable of causing only mild disease in WT mice. Therefore, the data shown here demonstrate that IFN-γ is produced early during infection and plays an important role in mediating host resistance to DENV-3 infection.

### IFN-γ-mediated protection to adapted-DENV-3 infection depends on enhanced NOS2-expression and nitric oxide production

**Figure 4:**
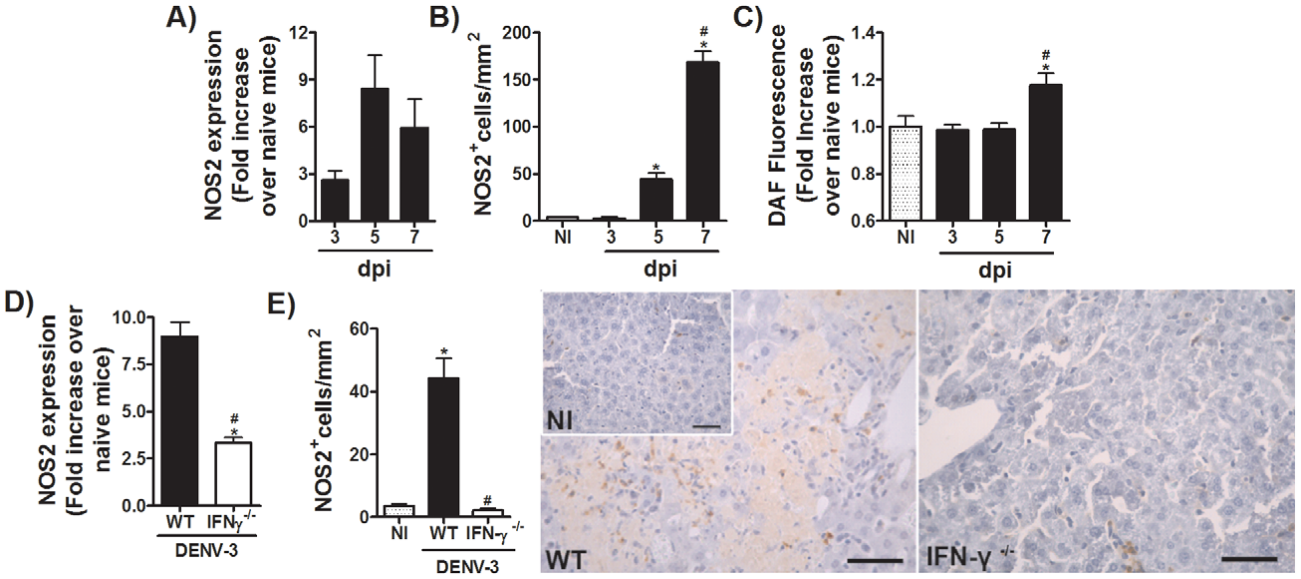
IFN-γ controls NOS2-mediated NO production during adapted-DENV-3 infection. (A–C) WT mice (*n* = 6 mice per group) were inoculated with 10LD_50_ (1000 PFU) of adapted-DENV-3 (i.p) and 3, 5 or 7 days after infection, mice were culled and tissue were collected for the following analysis: (A) Determination of NOS2 RNA expression by qPCR in spleen of control and DENV-3 infected mice. Results are shown as fold increase over basal expression in naive mice. (B) Determination of NOS2 staining by immunohistochemistry in liver sections of control and DENV-3 infected mice. Results are expressed as number of positive cells per mm^2^ of liver. (C) Esplenocytes were incubated with DAF-2DA and fluorescence determined. Results are expressed as fold increase in fluorescence over stained cells of naive mice. (D–E), WT and IFN-γ^−/−^ mice (n = 6 per group) were inoculated with 1LD_50_ (100 PFU) of DENV-3 (i.p) and in the fifth day of infection mice were culled and tissues were collected for the following analysis: (D) Determination of NOS2 RNA expression by qPCR in spleen of WT and IFN-γ^−/−^ DENV-3 infected mice. Results are shown as fold increase over basal expression in naive mice. (E) Determination of NOS2 staining by immunohistochemistry in liver sections of WT and IFN-γ^−/−^ DENV-3 infected mice. Results are expressed as number of positive cells per mm^2^ of liver. Results are expressed as mean ± SEM and are representative of at least two experiments. * for P<0.05 when compared to control naive mice. # for P<0.05 when compared to WT infected mice. 10LD_50_ corresponds to 1000 PFU of DENV-3. 1LD_50_ corresponds to 100 PFU of DENV-3. dpi – days post-infection. NI – Not-infected.

One of the well known effector mechanisms induced by IFN-γ after viral infections is enhancement of NOS2 expression in phagocytes. To assess the participation of this pathway in host response to dengue infection, we evaluated the kinetics of NOS2 expression and NO production after DENV-3 infection. As shown in Figure 4A, there was an increase in NOS2 mRNA expression in spleen starting on day 3 after DENV-3 inoculation and rising rapidly on days 5 and 7 post infection. In accordance with these data, WT infected mice showed increased NOS2-positive staining in liver from day 5 after infection, peaking at day 7 after infection (Figure 4B), virtually only in infiltrating leukocytes. In addition, there was elevation in DAF staining of esplenocytes isolated from DENV-3-infected mice, showing increased production of NO in spleen on day 7 post-inoculation (Figure 4C).

Consistently with the ability of IFN-γ to induce NOS2 mediated NO production, NOS2 mRNA expression in spleen was markedly decreased in IFN-γ^−/−^ mice (Figure 4D). Similarly, immunohistochemistry analysis revealed that NOS2 positive-cells were almost absent in liver on day 5 post-infection (Figure 4E). Furthermore, there was no production of NO by dendritic cells infected with DENV-3 *in vitro* (Figure S7). However, treatment of WT bone marrow derived dendritic cells with IFN-γ prior to DENV-3 infection resulted in production of significant amounts of NO, an effect that was absent in IFNGRI^−/−^ cells (Figure S7). These data suggest that NOS2-mediated NO production during DENV-3 infection is controlled by IFN-γ.

**Figure 5:**
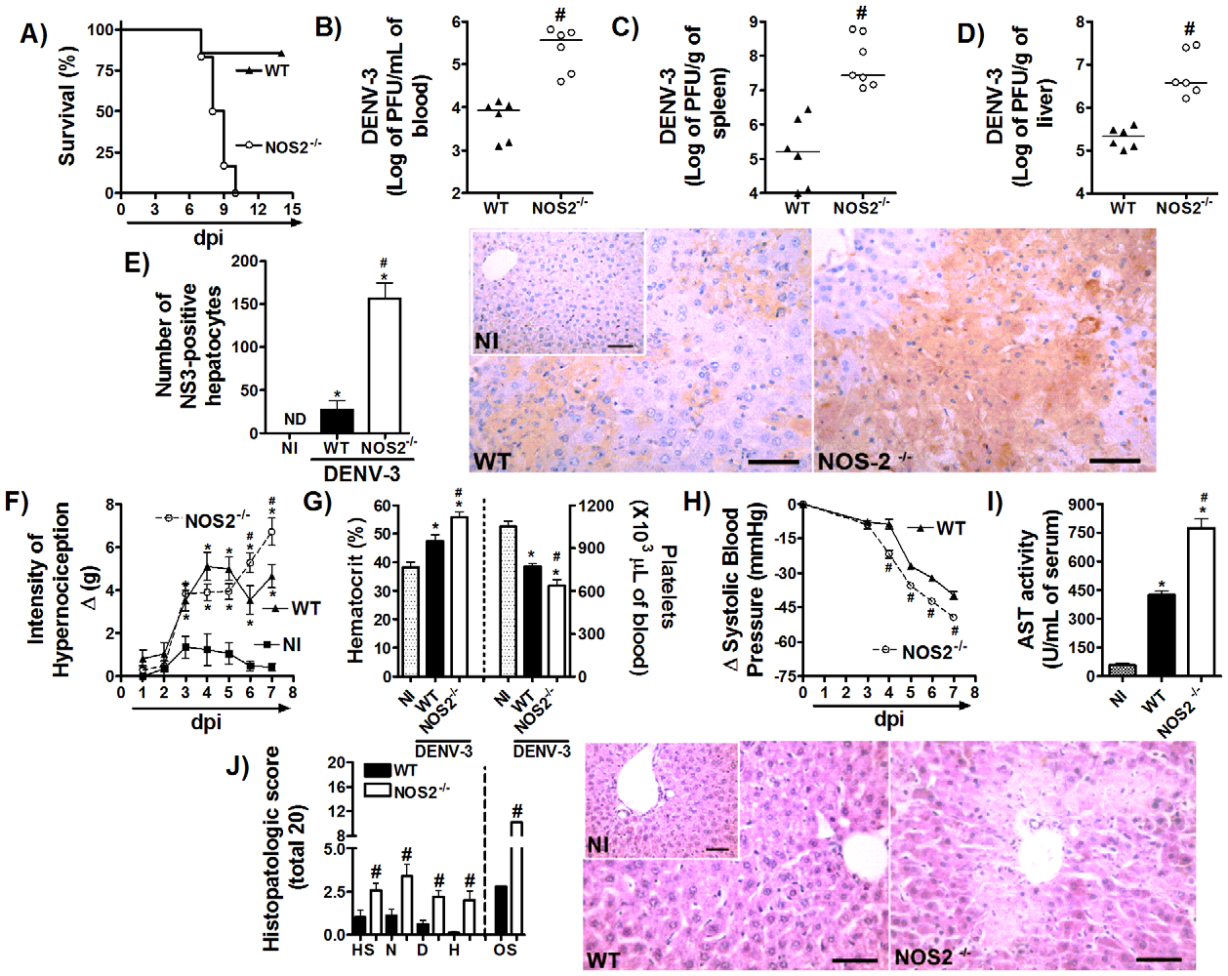
NOS2^−/−^ mice are more susceptible to adapted-DENV-3 infection. (A) WT and NOS2^−/−^ mice (n = 8 per group) were inoculated with 1LD_50_ (100 PFU) of DENV-3 (i.p) and lethality was evaluated every 12 hours during 14 days. Results are expressed as % of survival. In (B–J), WT and NOS2^−/−^ mice (n = 6 per group) were inoculated with 1LD_50_ (100 PFU) of DENV-3 (i.p) and in the seventy day of infection mice were culled and blood and tissues were collected for the following analysis: (B–D) Viral loads were recovered from the blood (B), spleen (C) and liver (D), respectively. Results are shown as the log of PFU per per mL of blood or g of tissue. (E) Serial sections from liver of WT and NOS2^−/−^ mice were stained with anti-DV NS3 antibody E1D8 (NS3) or an isotype control mouse IgG2a (IgG2a data not shown), and multiple sections of each tissue type were thoroughly examined for staining. Positive staining for NS3 is brown while hematoxylin counterstain is blue.Results are expressed as number of NS3-positive hepatocytes. (F) Mechanical hypernociception was assessed daily. Results are shown as the difference between the force (g) necessary to induce dorsal flexion of tibio-tarsal joint, followed by paw withdraw, before and after DENV-3 inoculation. In (G), hematocrit was shown as % volume occupied by red blood cells (left panel) and the number of platelets was shown as platelets ×10^3^/µl of blood (right panel). (H) Changes in Systolic blood pressure from baseline until day 5 after infection expressed as Δ of blood pressure in mmHg. In (I), AST activity determination in plasma was shown as U/dL of plasma. (J) shows semi-quantitative analysis of hepatic damage (histopathological analysis performed as modified from Paes et al, 2009) and Hematoxylin & Eosin staining of liver sections of control and WT and NOS2^−/−^ DENV-3-infected mice, seven days after infection. Scale Bar - 400 µm. The images presented are representative of an animal on the seventh day of infection. All results are expressed as mean ± SEM and are representative of at least two experiments. * for P<0.05 when compared to control uninfected mice. # for P<0.05 when compared to WT infected mice.1LD_50_ corresponds to 100 PFU of adapted-DENV-3. NI- Not-infected. dpi – days post-infection. HS – hepatocyte swelling. N – necrosis. D – degeneration. H – hemorrhage. OS – Overall Score.

To assess the role played by NOS2-induced NO during DENV infection, NOS2^−/−^ mice and their WT littermates were inoculated with 1LD_50_ of adapted DENV-3 and lethality rate and disease parameters were evaluated. As shown in Figure 5A, NOS2^−/−^ mice were markedly susceptible to DENV infection. While all knockout animals were dead by the 10^th^ day of infection, only 20% of WT mice had succumbed to infection after 14 days of inoculation of DENV-3. High viremia has been detected on day 5 after DENV-3 infection in NOS2^−/−^ mice (WT:7.8×10^3^ PFU/mL; NOS-2^−/−^: 8×10^5^ PFU/mL of blood, n = 6, p = 0.01). Viremia in NOS-2^−/−^ was also higher in comparison to WT littermates at day 7 post-DENV-3 inoculation (Figure 5B). Viral load in spleen (Figure 5C), and liver (Figure 5D) were also significantly higher in NOS2^−/−^ than in WT mice at day 7 post-DENV-3-inoculation. In addition, there was also increase in number of NS3-positive cells in liver in comparison with WT infected controls (Figures 5E). Importantly, NOS2^−/−^ mice showed significant mechanical hypernociception (Figure 5F), on days 6 and 7 after DENV-3 inoculation, in comparison with WT infected mice. NOS2^−/−^ also showed greater thrombocytopenia, intense hemoconcentration (Figure 5G right and left panels, respectively) and marked reduction in systolic blood pressure (Figure 5H) in comparison with WT infected mice. Finally, AST activity in serum was more intense in knockout mice in comparison to DENV-3 infected WT controls (Figure 5I). Similarly to the situation found in infected IFN-γ^−/−^ mice, important histopathological alterations were found in liver of NOS2^−/−^ mice after DENV-3 inoculation (Figure 5J). Histopathological analysis revealed greater disease scores in the NOS2^−/−^ group than in WT mice (Figure 5J). Of note, all alterations seen in NOS2^−/−^-infected mice were not due to a reduction in IFN-γ production after DENV-3 infection (for example, at day 7 in serum: NI = not-detectable; WT = 3530±317 pg/mL of serum; NOS2^−/−^ = 2968±619 pg/mL of serum, n = 6, p = 0,44).

After DENV-3 inoculation, there were detectable levels of both IL-12/23p40 and IL-18 cytokines in the spleen of WT mice already on day 5 of infection (Figure 1L). The early production of these cytokine is consistent with their possible role in the induction of IFN-γ during DENV-3 infection. As seen in the DENV-2 mouse model [30], there was drastic reduction in production of IFN-γ after DENV-3 infection in IL-12p40^−/−^ mice, which are deficient for both IL-12 and IL-23 cytokine production, and also in IL-18 binding protein (IL-18 bp) treated-WT mice (Figure S8A) or IL-18^−/−^ mice (NI = not-detectable; WT = 4435±562 pg/100 mg of spleen; IL-18^−/−^ = 2373±552 pg/100 mg of spleen, n = 6, p = 0,029). Interestingly, combined depletion of both cytokines resulted in total abrogation of IFN-γ staining in spleen cells after DENV-3 infection (Figure S8A). In accordance with these data, IL-12p40^−/−^ mice, IL-18^−/−^ mice or IL-12p40^−/−^ mice treated with IL-18 bp (IL12p40^−/−^+IL18 bp) were more susceptible to DENV-3 infection (Figure S8B). While only 20% of WT mice were dead at the end of 14 days after infection, all knockout mice had succumbed to infection until day 9 of DENV-3 inoculation. Significantly, earlier deaths were accompanied by elevation in viral loads in blood of IL-12p40^−/−^, IL-18^−/−^ or IL-12p40^−/−^ treated with IL18 bp mice (Figure S8C). Moreover, combined cytokine depletion resulted in intense hemoconcentration (Figure S8D, left panel), which was greater than in the other infected groups. Thrombocytopenia was also increased but there was no difference between the infected groups (Figure S8D, right panel).

## Discussion

Several important questions in dengue immunopathogenesis are difficult to address without adequate animal models of infection and disease. In the present study, we described a novel model of DENV-3 infection in adult immunocompetent mice which mimics the major manifestations of severe dengue infection in humans. We demonstrated that the inoculation of the mouse-adapted DENV-3 strain by a peripheral route induced an inoculum-dependent lethality preceded by severe disease development in adult immunocompetent mice. The major alterations found during disease development were: 1) Lethality preceded by development of hemoconcentration, thrombocytopenia, elevated transaminase levels associated with important liver injury, marked body weight loss and reduction in systolic blood pressure; 2) Increased levels of cytokines, including IFN-γ, IL-6, TNF-α, Il-12/23p40 and IL-18; 3) Increased viral load in spleen, liver and blood, virus NS1 antigen serum levels and NS3-staining in hepatocytes of infected mice. All these findings indicate that the mouse model described here will add to existing models [7], [16], [45]–[46] and together they may aid in the study of the immunopathogenesis of dengue disease.

The difficulty in developing a mouse model for DENV is largely the result of the inability of human clinical isolates to replicate well in mice [7], [16], [45], [47], [48]. Previously, our group has developed an experimental model of infection with an adapted DENV-2 strain that mimics several clinical parameters seen in human disease. This model has allowed the study of some mechanisms mediating protection or those associated with the development of severe disease [22], [24]–[28], [30]. One important key point facing dengue researchers is how a viral strain or serotype variation and infection sequence affects the conditions for immune protection and enhancement [48]. In this context, it becomes extremely necessary the establishment of primary and secondary mouse models with all DENV serotypes to verify if this mechanisms of protection and disease share similarities or differences in the face of different context of infections, and also to test the efficacy of possible vaccine candidates and antiviral compounds. Accordingly, using the same strategy described for the DENV-2 model [22] we generated an adapted strain of DENV-3 by several intracerebral (ICR) passages of a non-adapted human DENV-3 (genotype I) into weaning and progressively older BALB/c mice. Interestingly, this same non-adapted human strain was able to induce meningoencephalitis and behavioral changes that preceded lethality in adult C57BL/6 mice when inoculated by i.c route, however without causing any systemic clinical manifestation in these mice [49]. Importantly, the infection of immunocompetent mice with this new adapted-DENV-3 strain showed many similarities with the disease found in the DENV-2 infection model described before [22], [24] and also with the disease seen in humans. We believe that the characterization of these two DENV strains and the establishment of primary and secondary mouse models with all DENV serotypes will aid in evaluation of mechanisms of protection and of disease during distinct context of infections, as well as to test the efficacy of possible vaccine candidates and antiviral compounds.

Meanwhile, our studies demonstrated that after adaptation process, the adapted DENV-3 strain acquired the ability to induce systemic severe disease in adult immunocompentent mice (BALB/c or C57BL/6 strains) when inoculated by a peripheral route, without affecting the CNS, resembling the major manifestations seen in infected humans. It is important to note that the virus kept its ability to replicate in the CNS when given directly in the brain. Previous studies have also shown that the adaptation process is necessary for an efficient infection and occurrence of disease symptoms. Tesh and colls (2001) [50] showed that sequential series of liver-to-liver passages of YFVs in hamsters are necessary to lead the generation of more virulent strains. In addition, Shresta and colleagues (2004) [51] have generated a novel virulent DENV-2 strain, D2S10, by alternately passages between mosquito cells and non-neuronal tissues of mice. Using AG129 mice (that lack IFN type I and II receptors), they have demonstrated that D2S10 strain was more virulent than the parental strain, PL046, causing a lethal but nonparalytic disease. Sequence comparisons between D2S10 and the parental strain (PL046) revealed amino acid change difference in a conserved region of E gene, suggesting a role for these particular residues in determining viral virulence and pathogenesis *in vivo* [51]. Besides this, a full molecular analysis is necessary to identify the viral determinants responsible for this strong phenotype of dengue disease in the present model, but this is beyond the scope of the present study.

Hallmark features of human DHF/DSS are vascular leakage, higher viral burden, elevated levels of serum cytokines, hypotension and occurrence of thrombocytopenia [52]–[53]. Hence, all these features were observed in the present study, demonstrating that the present mouse model mimics severe dengue disease in humans. We, therefore, suggest that this model of dengue infection may be useful for the study of the pathophysiology of severe dengue disease. Epidemiological observations demonstrated that only a very small percentage of infections results in severe disease (DHF/DSS), represented as a tip of the pyramid and that this incidence varies significantly between primary and secondary DENV infections. It has been documented that a secondary DENV infection is the single and the most important risk factor for severe dengue disease manifestation, although, severe disease during primary infections is also reported [54], [55]–[59]. It has been hypothesized that subneutralizing levels of antibodies facilitate the entry of viral particles in permissive cells, enhancing viral loads, and exacerbating disease manifestation during secondary infection [60]. Experimental DENV models support this hypothesis and suggest that disease severity is directly associated with enhanced viral replication during infection [45]–[46]. In the present experimental model, lethality hematological and other pathological alterations in infected mice was dependent on the size of the inoculum and were observed in mice presenting elevated viral loads. Of note, infected IFN-γ-deficient and NOS2-deficient mice presented heightened viral replication, in parallel with elevated hematocrits, thrombocytopenia, and liver injury. Thus, although our studies do not mimic the human situation of 2 sequential infections with distinct viral serotypes, these results mimic up to the extent in which we demonstrated that disease in this model is inoculum-dependent, what bears relevance to human disease.

In addition to the features above, both clinical and experimental observations suggest that there is important liver involvement during dengue infection [61]–[63]. For example, elevated serum transaminase levels during dengue infection is common and is usually correlated with disease severity [8], [27], [43], [64]–[66]. In accordance, AST and ALT transaminase levels were elevated in the present study mainly on day 7 of infection, correlating with the peak of disease and hepatic damage in the present model. Further, intense necro-hemorrhagic hepatitis, hepatocellular swelling and steatosis associated with vascular damage, which is characteristic of dengue-induced hepatitis in human liver [64] were observed in liver of mice inoculated with a lethal inoculum of adapted-DENV3 on day 7 post-infection. Intravital confocal microscopy of the liver microvasculature revealed a significant increase in hepatocyte diameter and severe reduction in sinusoidal perfusion. In this sense, liver failure may be caused by reduction of sinusoidal perfusion (directly promoting tissue isquemia) and also by diffuse hepatocyte necrosis caused by DENV infection and replication and/or by products derived from inflammatory leukocytes. Our data on the liver injury showed many similarities to those demonstrated by Paes et al (2005, 2009) [43], [65], using a non-adapted DENV-2 in BALB/c mice and even to histopathological findings from DF and DHF postmortem tissue specimens [66]–[68]. Of note, we showed the presence of virus (or NS3 protein) in liver, mainly in hepatocytes. All these findings indicate the liver as an important target organ of DENV infection and replication, suggesting an important association between virus replication and hepatic damage as demonstrated by other studies [43], [45]–[46], [65], [67].

Its well knows that the IFN system is essential in the context of DENV infection [69]–[70]. Recently, we have demonstrated that optimal IFN-γ production during DENV-2 infection is controlled by the cytokines IL-12 and IL-18. Moreover, we showed that one of the mechanisms triggered by IFN-γ during host response to DENV-2 infection is the production of nitric oxide, an important virustatic metabolite. In this sense, to validate this novel mouse model of DENV-3 infection and to verify whether this pathway is also involved in response against different serotypes, we investigates the role of IFN-γ in the context of infection with the adapted-DENV-3 strain. In the present experimental model, IFN-γ is produced early (day five of infection) in infected-WT mice and the absence of IFN-γ action was associated with earlier lethality, more severe disease and higher viral loads even during infection with sublethal inoculums. These findings are in agreement with Shresta and coworkers (2004) [51] that demonstrated the importance of IFN-γ and type I IFNs in restricting viral replication and eliminating virus after primary DENV-2 infection. The correlation between increased IFN-γ production and higher survival rates in DHF patients also supports this idea [70]. Importantly, Gunther and colleagues (2011) [64] have demonstrated in a human challenge model of DENV infection that only sustained IFN-γ production was associated with protection against fever and viremia during the acute phase of illness [71]. In our studies, enhanced viral replication in IFN-γ-deficient mice was associated with more severe disease manifestation, as shown by enhanced hematological alterations and hepatic damage. These data strongly suggest that in the absence of IFN-γ, there are intense and uncontrolled viral replication, that lead to severe disease manifestation and lethality, already in early times of infection. Accordingly, previous studies have shown that IFN-γ likely contributes to viral clearance through several mechanisms, including direct inhibition of viral replication [72]. Of note, quite similar to that seen in the DENV-2 model [30], the combined action of IL-12 and IL-18 is also necessary for optimal IFN-γ production and control of DENV-3 infection.

Production of reactive nitrogen intermediates via increase in NOS2 expression is among the main IFN-γ-induced pathways involved in control of infections [73]. In fact, it has been shown that NOS2 expression is increased after DENV infection and that its expression in PBMCs of DF patients. This increase in NOS2 expression was found to correlate with the late acute phase of disease and preceded the clearance of DENV from monocytes [74]. NO production was also associated with less severe disease manifestation disease in humans [75]. Finally, NO is able to inhibit DENV replication *in vitro* [76]–[77]. In the present study, NOS2 expression is increased during adapted DENV-3 infection in different targets organs of infection and this expression was controlled by IFN-γ. Nitric oxide production was also observed in esplenocytes of DENV-3 infected mice (*ex vivo)* and in DENV-infected DCs stimulated by IFN-γ *in vitro*. Of note, NOS2^−/−^ mice had elevated lethality, more severe disease manifestation and increased viral loads, even in the presence of high levels of IFN-γ. Thus, these data demonstrated here show that NOS2-mediated NO production after primary DENV-3 infection also seems to be an important pathway involved in control of DENV-3 replication and disease evolution. These data are quite similar with the results found in the DENV-2 infection model, suggesting that this mechanism is conserved protective pathway in host response to both DENV-2 and DENV-3 serotypes. These findings support that strategies aiming to potentiate IFN-γ-induced NO production could be useful during the control of primary infection by Dengue virus.

In summary, we report a model of DENV-3 infection in immunocompetent mice and describe the clinical, immunopathological and virological features induced by inoculation of the virus. These features clearly resemble the manifestations of severe dengue disease in humans. We have also demonstrated the crucial role of IFN-γ and NOS2-derived NO in host resistance to DENV infection, a protective pathway involved in resistance to other DENV strains. Therefore, this model represents a significant advance in animal models of severe dengue disease and may contribute to the elucidation of the immunopathogenesis of disease and of protective mechanisms associated to infection. In addition, the model may be a relevant tool for vaccine and drug development.

## Supporting Information

Figure S1 **Disease parameters in BALB/c mice infected with an adapted strain of DENV-3.** (A) WT mice (*n* = 6 mice per group) were inoculated with different inoculums of adapted-DENV-3 (i.p) and lethality was evaluated every 12 hours for 14 days. Results are expressed as % of survival. In Figs (B–E) WT mice (n = 6 per group) were inoculated with 1LD_50_ (100 PFU) of DENV-3 (i.p) and in the third, fifth or in the seventh day of infection mice were culled and blood and tissues were collected for the following analysis: (B) Change in body weight was expressed as percentage of initial weight loss. (C) Mechanical hypernociception was assessed daily. Results are shown as the difference between the force (g) necessary to induce dorsal flexion of tibio-tarsal joint, followed by paw withdraw, before and after DENV-3 inoculation. (D) Hematocrit was expressed as % volume occupied by red blood cells (left panel) and the number of platelets was shown as platelets ×10^3^/µl of blood (right panel) and. In (E) AST and ALT activity determination in plasma of control and DENV-3-infected mice was shown as U/dL of plasma. Results are expressed as mean ± SEM and are representative of at least two experiments. * for P<0.05 when compared to control uninfected mice. 1 LD_50_ corresponds to 100 PFU of DENV-3. NI – not-infected. dpi- days post-infection.
(TIF)

Figure S2 **Confocal microscopy in liver microvasculature upon DENV-3 infection.** (A-B) C57BL/6j mice (n = 5 per group) were inoculated with 10LD_50_ (1000 PFU) of DENV-3 (i.p) and in the seventh day of infection mice were anesthetized, cells were fluorescently labeled by rhodamine 6G (A) or phycoerythrin-anti PECAM-1 antibody (B) to assess hepatocyte size and the percentage of perfused sinusoids, respectively. Hepatocyte size is expressed as the length of longest cell axis and perfused sinusoids as % of the area fraction stained by the antibody. The images presented are representative of an animal on the seventh day of infection.All results are expressed as mean ± SEM and are representative of at least two experiments. * for P<0.05 when compared to control uninfected mice. 10 LD_50_ corresponds to 1000 PFU of adapted-DENV-3. dpi- days post-infection. NI: Not infected.
(TIF)

Figure S3 **Viral load and lethality rates of BALB/c mice upon adapted-DENV-3 inoculation.** (A–C) WT mice and their controls (n = 6-7 per group) were inoculated with 1LD_50_ (100 PFU) of DENV-3 (i.p) and in the third, fifth or in the seventh day of infection mice were culled and blood and tissues were collected for the following analysis: (A–C) Viral loads were recovered from the spleen, liver and blood, respectively. Results are shown as the log of PFU per g of tissue or per mL of blood. In (D), plaque purification technique was performed for isolation of DENV-3 clones (Clone 1–9) and mice were inoculated with 100 PFU of each clone and lethality was evaluated every 12 hours for 14 days. Results are expressed as % of survival. * for P<0.05 when compared to control uninfected mice. 1 LD_50_ corresponds to 100 PFU of adapted-DENV-3. NI – not infected. ND- not detectable. dpi- days post-infection.
(TIF)

Figure S4 **Typing of adapted DENV-3.** (A) Agarose gel electrophoresis displaying amplicons of specific PCR with primers D1 and TS3 (290 bp). Lane 1, DNA Ladder **50** bp, Lane 2, empty. Lane 3: Non-adapted DENV-3 (positive control). Lane 4-6: adapted DENV-3 (several passages in C6/36 cells). Lane 7: primer mix. (B) Polyacrylamide gel electrophoresis displaying amplicons of specific PCR with primers D1 and TS3 (290 bp). Lane 1, Clone 4 of the adapted DENV-3, Lane 2, DNA Ladder **50** bp. Lane 3: primer mix. Lane 4: Non-adapted DENV-3 (positive control).
(TIF)

Figure S5 **Inoculation of Clone 4 in C57BL/6j mice mimics the disease and mortality seen after infection with adapted-DENV-3.** (A) C57BL/6j mice (*n* = 5 mice per group) were inoculated with different inoculums of plaque purified Clone 4 (i.p) and lethality was evaluated every 12 hours for 14 days. Results are expressed as % of survival. In Figs (B–D) C57BL/6j mice (n = 6 per group) were inoculated with 10LD_50_ (100 PFU) of Clone 4 (i.p) and in the third, fifth or in the seventh day of infection mice were culled and blood were collected for the following analysis: (B) Viral load was recovered from the blood. Results are shown as the log of PFU per mL of blood. (C–D) The number of platelets was shown as platelets ×10^3^/µl of blood (C) and hematocrit as % volume occupied by red blood cells (D). * for P<0.05 when compared to control uninfected mice. 10LD_50_ corresponds to 100 PFU of Clone 4. NI – not infected. ND- not detectable. dpi- days post-infection.
(TIF)

Figure S6 **Virus analysis in brain and spleen of newborn BALB/c mice upon adapted-DENV-3 i.p and i.c inoculation.** BALB/c newborn mice (n = 4–7 per group) were inoculated with 20 PFU of adapted-DENV-3 by i.c or i.p route and 5 days after infection spleen and brain were collected for the following analysis: (A–B) Viral loads were recovered from the brain and spleen, respectively. Results are shown as the log of PFU per g of tissue. (C) Semiquantitative analysis of multiple sections of brain samples from i.c or i.p DENV-3 inoculated mice on day 5 after infection. Results are shown as number of positive cells. * for P<0.05 when compared to control uninfected mice. # for P<0.05 when compared to i.c newborn infected mice. i.c – intracerebral. i.p – intraperitoneal. NI – not infected. ND – Not detectable.
(TIF)

Figure S7 **Enhanced NO production by DCs after adapted-DENV-3 infection is controlled by IFN-γ.** Bone marrow derived dendritic cells were infected with DENV-3 (MOI 0,05 PFU/cell) in the presence or not of IFN-γ. After 72 hours, cell supernatant was collected for nitrite quantification by Griess reaction. Results are expressed as and µM of nitrite in medium. Results are expressed as mean ± SEM and are representative of at least two experiments. * for P<0.05 when compared to control uninfected cells, and # for P<0.05 when compared to DENV-3-infected cells.
(TIF)

Figure S8 **IL-12 and IL-18 act in synergism to induce IFN-γ production and resistance to DENV-3 infection.** (A) WT and IL-12/p40^−/−^ mice (*n* = 4 mice per group) treated or not with IL-18 bp (daily i.p. injection of 1 mg/kg of protein) were inoculated with 1LD_50_ (100 PFU) of DENV-3 (i.p), culled on day seven of infection and esplenocytes isolated for assaying IFN-γ production by cellular staining with labeled antibodies and FACS analysis. Results are expressed as % of IFN-γ-positive cells in each population. (B) WT, IL-12/p40^−/−^ mice (*n* = 6 mice per group) treated or not with IL-18 bp (daily i.p. injection of 1 mg/kg of protein) and IL-18^−/−^ were inoculated with 1LD_50_ (100 PFU) of DENV-3 (i.p) and lethality was evaluated every 12 hours for 14 days. Results are expressed as % of survival. (C, D) WT and IL-12/p40^−/−^ mice (*n* = 4 mice per group) treated or not with IL-18 bp (daily i.p. injection of 1 mg/kg of protein) were inoculated with 1LD_50_ (100 PFU) of DENV-3 (i.p), culled on day seven of infection and tissues collected for (C) Viral load quantification in blood of WT, IL-12p40 treated or not with IL-18 bp (daily i.p. injection of 1 mg/kg of protein) and IL-18^−/−^ upon DENV-3 infection Results are shown as the log of PFU per mL of blood; (D) Hematocrit measurement, shown as % volume occupied by red blood cells (left panel) and number of platelets shown as platelets ×10^3^/µl of blood (right panel). Results are expressed as mean ± SEM (except for C, expressed as median) and are representative of at least two experiments. * for P<0.05 when compared to control uninfected mice. # for P<0.05 when compared to WT infected mice. 1 LD_50_ corresponds to 100 PFU of DENV-3. NI – not-infected.
(TIF)
