# Supplementary figures and images for: A Model of DENV-3 Infection That Recapitulates Severe Disease and Highlights the Importance of IFN-γ in Host Resistance to Infection
Source: PLoS Negl Trop Dis. 2012 May 29;6(5):e1663. doi: 10.1371/journal.pntd.0001663 (PMC3362616; doi:10.1371/journal.pntd.0001663)

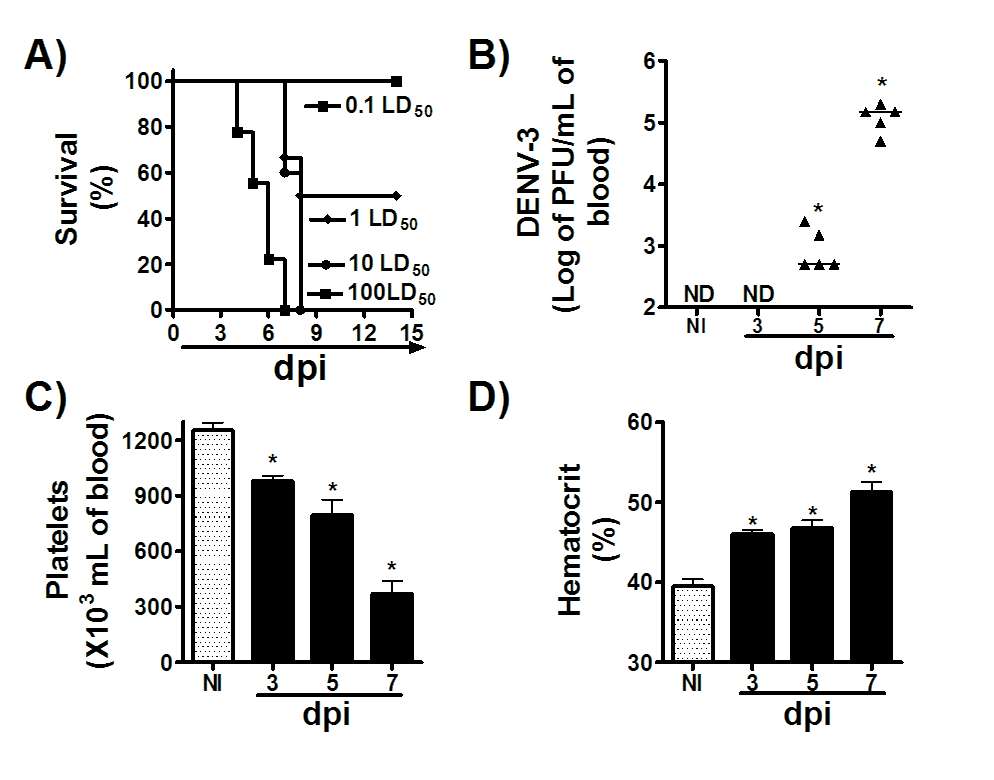

Supplement: Figure S5 — Inoculation of Clone 4 in C57BL/6j mice mimics the disease and mortality seen after infection with adapted-DENV-3. (A) C57BL/6j mice (n = 5 mice per group) were inoculated with different inoculums of plaque purified Clone 4 (i.p) and lethality was evaluated every 12 hours for 14 days. Results are expressed as % of survival. In Figs (B–D) C57BL/6j mice (n = 6 per group) were inoculated with 10LD50 (100 PFU) of Clone 4 (i.p) and in the third, fifth or in the seventh day of infection mice were culled and blood were collected for the following analysis: (B) Viral load was recovered from the blood. Results are shown as the log of PFU per mL of blood. (C–D) The number of platelets was shown as platelets ×103/µl of blood (C) and hematocrit as % volume occupied by red blood cells (D). * for P<0.05 when compared to control uninfected mice. 10LD50 corresponds to 100 PFU of Clone 4. NI – not infected. ND- not detectable. dpi- days post-infection. (TIF) [file pntd.0001663.s005.tif]

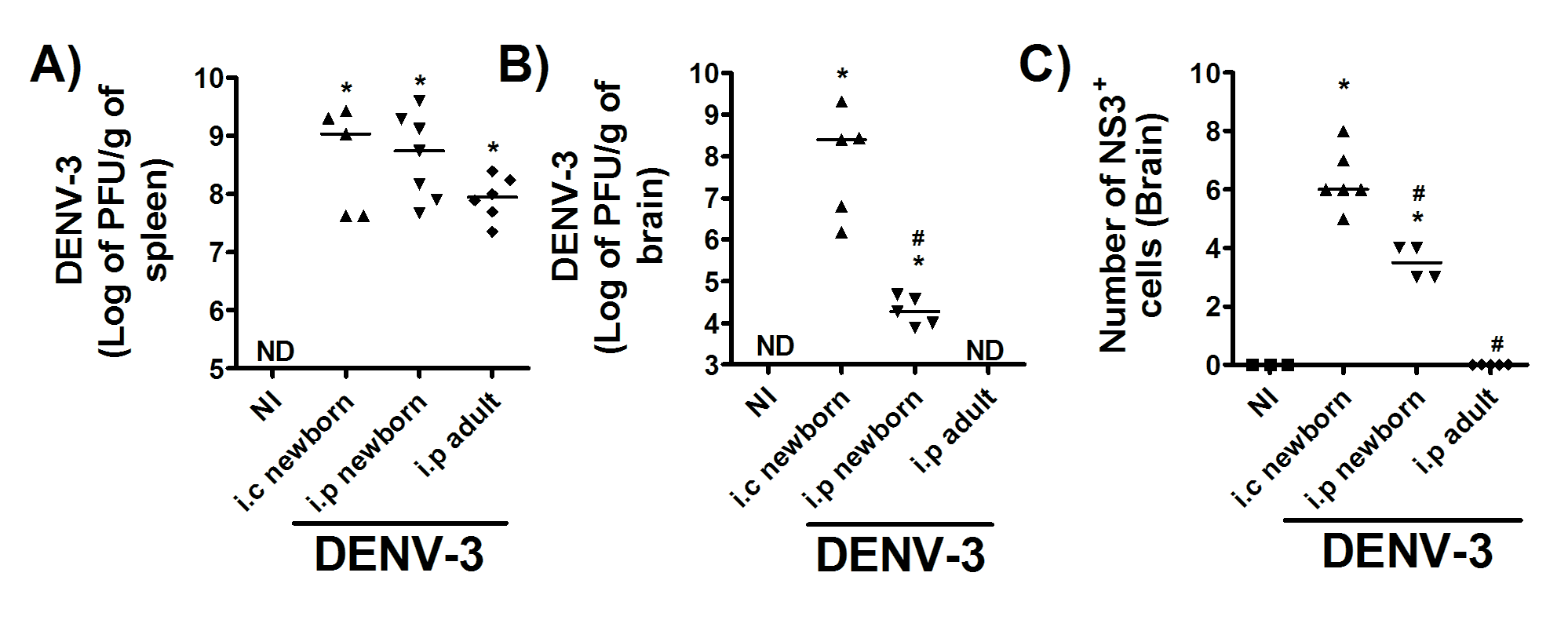

Supplement: Figure S6 — Virus analysis in brain and spleen of newborn BALB/c mice upon adapted-DENV-3 i.p and i.c inoculation. BALB/c newborn mice (n = 4–7 per group) were inoculated with 20 PFU of adapted-DENV-3 by i.c or i.p route and 5 days after infection spleen and brain were collected for the following analysis: (A–B) Viral loads were recovered from the brain and spleen, respectively. Results are shown as the log of PFU per g of tissue. (C) Semiquantitative analysis of multiple sections of brain samples from i.c or i.p DENV-3 inoculated mice on day 5 after infection. Results are shown as number of positive cells. * for P<0.05 when compared to control uninfected mice. # for P<0.05 when compared to i.c newborn infected mice. i.c – intracerebral. i.p – intraperitoneal. NI – not infected. ND – Not detectable. (TIF) [file pntd.0001663.s006.tif]

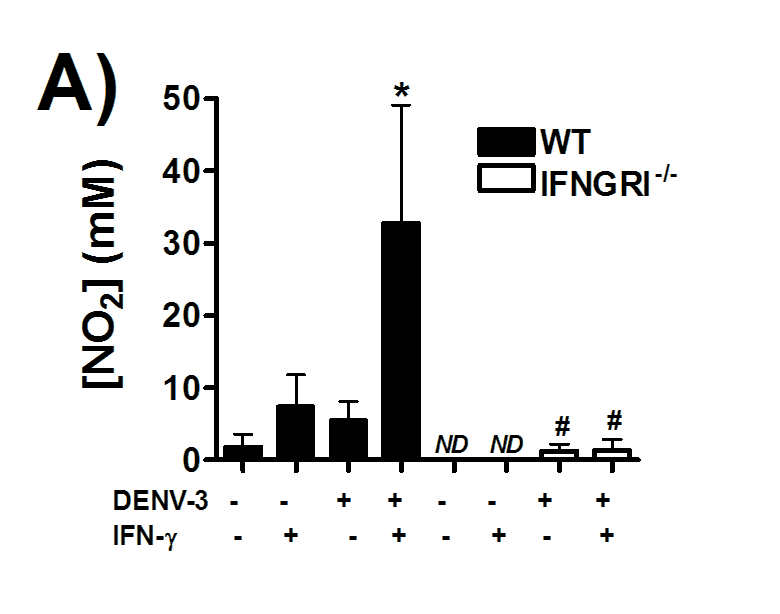

Supplement: Figure S7 — Enhanced NO production by DCs after adapted-DENV-3 infection is controlled by IFN-γ. Bone marrow derived dendritic cells were infected with DENV-3 (MOI 0,05 PFU/cell) in the presence or not of IFN-γ. After 72 hours, cell supernatant was collected for nitrite quantification by Griess reaction. Results are expressed as and µM of nitrite in medium. Results are expressed as mean ± SEM and are representative of at least two experiments. * for P<0.05 when compared to control uninfected cells, and # for P<0.05 when compared to DENV-3-infected cells. (TIF) [file pntd.0001663.s007.tif]
